# Evolution of educational inequalities in site-specific cancer mortality among Belgian men between the 1990s and 2000s using a “fundamental cause” perspective

**DOI:** 10.1186/s12885-017-3461-8

**Published:** 2017-07-05

**Authors:** Katrien Vanthomme, Hadewijch Vandenheede, Paulien Hagedoorn, Sylvie Gadeyne

**Affiliations:** Interface Demography, Department of Social Research, Faculty of Economic and Social Sciences and Solvay Business School, Vrije Universiteit Brussel, Pleinlaan 2, 1050 Brussels, Belgium

**Keywords:** Cancer, Mortality, Socioeconomic inequality, Fundamental cause theory

## Abstract

**Background:**

According to the “fundamental cause” theory, emerging knowledge on health-enhancing behaviours and technologies results in health disparities. This study aims to assess (trends in) educational inequalities in site-specific cancer mortality in Belgian men in the 1990s and the 2000s using this framework.

**Methods:**

Data were derived from record linkage between the Belgian censuses of 1991 and 2001 and register data on mortality. The study population comprised all Belgian men aged 50–79 years during follow-up. Both absolute and relative inequality measures have been calculated.

**Results:**

Despite an overall downward trend in cancer mortality, educational differences are observed for the majority of cancer sites in the 2000s. Generally, inequalities are largest for mortality from preventable cancers. Trends over time in inequalities are rather stable compared with the 1990s.

**Conclusions:**

Educational differences in site-specific cancer mortality persist in the 2000s in Belgium, mainly for cancers related to behavioural change and medical interventions. Policy efforts focussing on behavioural change and healthcare utilization remain crucial in order to tackle these increasing inequalities.

## Background

The aim of this paper is to unravel educational inequalities in male cancer mortality in Belgium and to gain an insight into the evolution of these inequalities between the 1990s and the 2000s. Socioeconomic position (SEP) and, hence, education as an indicator of SEP is associated with many causes of death, including cancer [1–5]. Socioeconomic (SE) gradients in cancer mortality and the evolution in these gradients from one point in time to another vary by cancer site [1, 6]. To explain possible underlying mechanisms of these patterns, Link and Phelan’s fundamental cause theory (FCT) offers an interesting framework.

### Fundamental cause theory (FCT) and socioeconomic inequality

According to Link and Phelan, a fundamental social cause of health inequalities has four essential features [7, 8]: *(i)* it influences multiple disease outcomes; *(ii)* it affects disease outcomes through multiple risk factors; *(iii)* the association with health inequalities is reproduced over time; and *(iv)* it involves resources that can be used to minimize the risk of the disease.

SEP inequalities in health are created and reproduced through a growing ability to control disease and death, within the context of existing social and economic inequalities [9–11]. When new knowledge on health-enhancing behaviours or new medical interventions arises, the benefits of these developments are not distributed equally throughout the population [9, 11]. Those with greater access to resources such as knowledge, money, power, prestige and beneficial social connections will be more likely to use these new mechanisms to their health advantage and, hence, to experience lower mortality [7–9, 11–13]. Because of the flexible nature of these resources, they can be used no matter what the risk and protective factors are at play at a particular place and time [14]. Moreover, these resources operate both at the individual (health-enhancing behaviours) and contextual levels (risk profiles of neighbourhoods, occupations, and social networks) [8, 13, 15].

### Fundamental cause theory (FCT) and cancer mortality

From this point of view, SEP is indisputably a fundamental cause of cancer mortality. Research has shown that mortality from preventable and treatable cancers is more strongly related to SEP than mortality from non-preventable and non-treatable cancers [7, 9, 14, 16–19]. These inequalities in site-specific cancer mortality follow a specific pattern over time, depending on the advancements that are made on the knowledge about risk and protective factors of cancer, as well as in treatments [9, 14]. When there is sound knowledge of the causes and cures of cancers, those with greater access to resources or those situated in high SEP contexts, will disproportionally benefit from these advances [6, 7]. Knowledge may become available earlier and may be distributed faster within these high SEP contexts [6]. A perfect example is the association between smoking and lung cancer. At the beginning of the smoking epidemic, people with high SEP were more likely to smoke. Yet, with the development and dissemination of knowledge on the causal link of smoking with lung cancer in the 1950s and 1960s, the association with SEP reversed: smoking became more common among people of low SEP [6, 20]. Consequently, while lung cancer mortality used to be higher in high-SEP versus low-SEP individuals, it is currently more common in low-SEP groups. Low SEP is associated with multiple behavioural risk factors for cancer (mortality), such as smoking, alcohol abuse, lack of exercise, poor diet, overweight, unsanitary living conditions, and occupational hazards [7, 9, 12]. Moreover, for some cancers, such as colorectal cancer, survival chances can increase because of screening, early detection and timely treatment [21]. However, there are persistent SEP differences in access to and quality of healthcare, stage at diagnosis, and screening rates [8, 21–24].

### Research aims

The aims of the paper are twofold. The first aim is to assess educational inequalities in mortality from different cancer sites in Belgian men in the 2000s. Based on the FCT, we hypothesize that educational inequalities will be in favour of high educated men for several cancer sites and especially for *preventable* cancers (such as cancer of the head and neck, oesophagus, and lung). Secondly, the evolution of these inequalities between the 1990s and the 2000s will be investigated. We hypothesize that educational inequalities will have increased over time for preventable cancer sites because we assume that there is still an uneven distribution of health-beneficial innovations in society. This study will enable us to identify the cancer sites with the largest inequalities and the largest changes in inequalities during the last decades.

International research has shown diverging trends by gender [1], so focus should best be on men and women separately. Therefore, this paper focuses on Belgian men.

This study is unique in its focus on multiple cancer sites and on time trends in SE inequalities in cancer mortality. Such study design sheds light on the association between SEP and cancer and on the causes of these SEP inequalities in cancer mortality and allows for a better understanding of SE inequalities in general. In Belgium in particular, studies investigating (time trends of) SE inequalities in cancer mortality are scant. As Belgium has among the highest cancer mortality rates in Europe, especially for breast and lung cancer [25], it constitutes a relevant setting to understand the patterning of SE inequalities in cancer mortality. Moreover, Belgian mortality data are quite unique outside the Nordic context, since they have population coverage, including all cancer deaths in Belgian inhabitants for the study period.

## Methods

### Design and study population

Data were derived from record linkage between the Belgian censuses of 1991 and 2001 and register data on mortality and emigration. In a first stage, a link was established between the 1991 census and register data concerning all deaths and emigrations during 01/03/1991–31/12/1997 and between the 2001 census and emigration/mortality data during 01/10/2001–31/07/2008. In a second stage, cause-specific mortality data have been added using anonymous individual linkage with death certificates. The database is a unique source of information containing data on mortality, emigration, causes of death, and background characteristics of all individuals legally residing in Belgium at the time of the 1991 and 2001 census.

The study population comprised all Belgian male inhabitants aged between 50 to 79 years during the follow-up period. Subjects older than 79 years were excluded from the analyses, because of the high proportion of missing data in the older age groups (e.g. 17% missing for education), and because cause-specific mortality analyses are more difficult to interpret at older age due to the increasing number of comorbidities [1, 16]. Men younger than 50 years were also excluded from the analyses since they die from other causes of death compared to older men.

### Variables

All cancer sites representing at least 1 % of total cancer mortality (i.e. more than 1000 cases) in one of the follow-up periods were included in the analyses. The cancer sites were defined following the International Classification of Diseases and Related Health Problems (ICD). For mortality in the 1990s, ICD-9 was used; for mortality in the 2000s, ICD-10 was used. To classify the cancers by level of preventability, we used the often applied criteria developed by Mackenbach and colleagues [16]: *amenability to behavioural change* and *amenability to medical interventions*. Cancer sites were operationalized as *amenable to behavioural change* if the combined population attributable fraction (PAF) of mortality for overweight and obesity, low fruit and vegetables intake, physical inactivity, unsafe sex, smoking and alcohol use was larger than 50% for European men in the Global Burden of Disease and Risk Factors study [26]. According to the definition of Mackenbach, cancer sites were operationalized as *amenable to medical interventions* if the 5-year relative survival rate for Belgian men during 2000–2007 was higher than 70% in the EUOCARE project [27]. Additionally in this study, we operationalized cancer sites for which effective screening programmes are available in Belgium [28] as amenable to medical interventions. The cancer sites studied in this paper, their corresponding ICD-codes and their level of preventability are listed in Table 1.

**Table 1:** International Classification of Diseases (ICD-) codes for the cancer sites included in the analysis (9th and 10th revision) and level of preventability

	ICD-9	ICD-10	Behavioural change	Medical interventions
Malignant neoplasms of:				
Head and neck	140–149, 160–161	C00-C14, C30-C32	Yes	No
Oesophagus	150	C15	Yes	No
Stomach	151	C16	Yes	No
Colorectum and anus	153–154	C18-C21	No	Yes
Liver	155	C22	Yes	No
Pancreas	157	C25	No	No
Lung, bronchus and trachea	162	C33-C34	Yes	No
Prostate	185	C61	No	Yes
Kidney	189	C64-C66, C68	No	No
Bladder	188	C67	No	Yes
Eye, brain and central nervous system	190–192	C69-C72	No	No
Malignant melanoma	172–173	C43-C44	No	Yes
Non-Hodgkin Lymphoma	200, 202	C82-C85	No	No
Multiple myeloma	203	C90	No	No
Leukaemia	204–208	C91-C95	No	No

Education was used as an indicator of SEP. Education is acknowledged as a good indicator of SEP for several reasons: it is completed early in life and therefore less prone to selection problems; it is stable over time; and it is available for nearly everyone in the population, contrary to, for example, occupation [16, 19, 29]. Educational attainment was categorized according to the International Standard Classification of Education (ISCED), version 1997: lower secondary education or less (ISCED 0–2; “low”), higher secondary education (ISCED 3–4; “mid”), and tertiary education (ISCED 5–6; “high”). Age was introduced as a time varying variable to account for age changes during the 8-year follow-up period. To do so, individual’s follow-up time was split into episodes each corresponding to different 5-year attained age groups [30].

### Statistical analyses

Both absolute and relative inequality measures of inequality were calculated to obtain the full picture of inequality patterns in cancer mortality [31–34].

Absolute inequalities consisted of the difference between calculated age-standardized mortality rates (ASMRs) by education. Age-standardized mortality rates and their 95% Confidence Intervals (95% CI) were calculated by period and educational level using the Belgian population at the time of the 2001 census as standard population. The absolute mortality rate difference (MRD) was measured as the difference between the ASMR of the lowest educated group and the ASMR of the highest educated group. To gain an insight into trends in absolute inequalities, we calculated the absolute and proportional mortality declines, based on the ASMR. These measures are the result of subtracting the absolute/proportional mortality decline among the high educated from the absolute/proportional mortality decline among low educated men [35]. Furthermore, we calculated the population-attributable fractions (PAF) of education for site-specific cancer mortality in the two periods. This measure reflects the proportional reduction (or increase) of population mortality that would occur if the total population had the same mortality rates as the high educated group. This measure provides relevant information from a public health point of view [1].

To calculate relative inequalities, age-adjusted mortality rate ratios (MRRs) were calculated using Poisson regression, comparing the “low” and “mid” educated versus the “high” educated. To assess the trend over time in relative inequalities, relative indices of inequality (RII) were calculated. The RII is based on a rank variable that calculates the mean proportion with a higher level of education for each educational group. This rank variable is then regressed on site-specific cancer mortality, using age-adjusted Poisson regression while adjusting for age. The resulting RII expresses inequality within the whole educational continuum and can be interpreted as the ratio of the mortality rates of the lowest versus the highest educated. Since the RII accounts for the educational distribution, comparing populations with different educational distributions is highly suitable, on the condition that there is a linear association between education and cancer mortality [1, 2, 4]. When the association between education and mortality was non-linear, RIIs were not presented. The significance of the trend over time was formally tested as explained by Altman & Bland [36].

As there is a strong association between region and mortality in Belgium [37], all Poisson models were adjusted for region (Flanders, Wallonia and Brussels). Moreover, health differences have been observed according to migration history [38], therefore migrant background (native versus non-native) was also added as a control variable in the analyses.

Education was operationalized using three categories. Consequently, the results are based on differences between three broad groups. To test the robustness of the results, a sensitivity analysis was conducted using a four-category classification for education, distinguishing primary education or less (ISCED 0–1) from those with lower secondary education (ISCED 2), leading to similar inequality patterns. In addition, housing tenure was used as an alternative indicator of SEP, leading to similar results (based on the ASMRs and MRRs).

Cases with missing information on educational attainment (8.5% in the 1990s and 9.5% in the 2000s) were excluded from the analyses. A sensitivity analysis was conducted including the cases with missing education data as a separate category. In general, for the 1990s, this did not yield different results whereas in the 2000s, men with a missing value on the educational variable showed higher mortality rates for overall cancer mortality as well as for the majority of the cancer sites. This probably implies a conservative bias, underestimating the association between education and cancer mortality. All analyses were performed using STATA 13.1.

## Results

### Description of the study population

The study population consisted of all Belgian men aged 50–79 years during the follow-up periods of 1991–1997 and 2001–2008. Due to the ageing of the population, the share of the 50- to 79-year-olds was larger in the most recent period, i.e. 39% compared to 35% (Table 2). During the 1990s and 2000s observation periods, 76,117 and 70,181 men died of cancer respectively. The educational distribution differed between both periods, with a larger percentage of low educated men in the 1990s than in the 2000s (73% compared to 57%) and a smaller percentage of high educated men (11% compared to 21%).

**Table 2:** Descriptive statistics for the Belgian male study population aged 50–79 years for the periods 1991–1997 and 2001–2008

	1991–1997	2001–2008
Number of men aged 50–79 years during follow-up	1,714,999	1,967,404
Percentage of the total male population aged 50–79 years during follow-up	35.17%	39.28%
Person-years	9,048,137	10,362,159
Number of cancer deaths	76,117	70,181
Distribution of education (%)		
Lower secondary or less	73.14	56.68
Upper secondary	15.26	21.92
Tertiary education	10.65	21.40

Source: censuses of 1991 and 2001 linked to Population and Mortality Register Data for the periods 1991–1997 and 2001–2008

### Are there educational inequalities in cancer mortality for Belgian men in the 2000s?

Total cancer mortality showed a clear gradient in the period 2001–2008 both in absolute and relative terms (Table 3). As for absolute inequalities, the mortality rate difference (MRD) between low-and high-educated men for all cancer mortality was 238.4 (95% CI: 234.1–242.7) deaths per 100,000 men in the 2000s. The majority of the *preventable* cancers showed educational inequalities in mortality. For all cancer sites related to *behavioural change*, mortality did vary by educational attainment. The largest educational inequality was observed for lung cancer, with a MRD of 151.7 (95% CI: 150.2–153.2) deaths per 100,000 men. For the cancer sites amenable to *medical interventions*, we observed lower mortality rates for high educated men in the 2000s for colorectal and bladder cancer only.

**Table 3:** Absolute and relative educational inequalities in site-specific cancer mortality among Belgian men aged 50–79 years for the period 2001–2008

Cancer site		Total population	Low educated ^c^	Mid educated ^d^	High educated ^e^	MRD ^f,g^ (95% CI ^h^)
Total cancer mortality	ASMR ^a^	687.0 (682.0–692.1)	729.8 (722.8–736.8)	612.6 (600.5–624.6)	491.4 (480.1–502.7)	238.4 (234.1–242.7)
	MRR ^b^		1.55 (1.52–1.59)	1.29 (1.25–1.33)	1.00	
Head and neck	ASMR	30.7 (29.6–31.7)	33.4 (31.8–34.9)	26.4 (24.0–28.8)	18.7 (16.6–20.7)	14.7 (14.2–15.2)
	MRR		1.95 (1.74–2.19)	1.50 (1.31–1.72)	1.00	
Oesophagus	ASMR	24.6 (23.6–25.5)	24.9 (23.6–26.2)	25.1 (22.7–27.5)	21.4 (19.1–23.7)	3.5 (2.5–4.5)
	MRR		1.26 (1.12–1.42)	1.24 (1.08–1.43)	1.00	
Stomach	ASMR	22.0 (21.1–22.9)	23.8 (22.5–25.0)	17.2 (15.1–19.2)	16.1 (14.0–18.1)	7.7 (6.9–8.5)
	MRR		1.50 (1.31–1.72)	1.09 (0.92–1.29)	1.00	
Colorectal	ASMR	65.9 (64.3–67.4)	66.2 (64.1–68.3)	63.2 (59.3–67.1)	52.4 (50.4–58.0)	13.8 (10.3–13.7)
	MRR		1.25 (1.16–1.34)	1.19 (1.09–1.30)	1.00	
Liver	ASMR	19.5 (18.6–20.3)	18.8 (17.7–20.0)	20.7 (18.5–22.9)	18.9 (16.7–21.2)	−0.1 (−1.2–1.0)
	MRR		1.10 (0.96–1.25)	1.20 (1.03–1.41)	1.00	
Pancreas	ASMR	33.2 (32.0–34.3)	33.8 (32.3–35.3)	31.3 (28.5–34.0)	33.5 (30.5–36.5)	0.3 (−1.2–1.8)
	MRR		1.07 (0.97–1.18)	0.98 (0.87–1.11)	1.00	
Lung and trachea	ASMR	252.9 (249.9–256.0)	286.1 (281.7–290.5)	196.7 (189.9–203.6)	134.4 (128.5–140.3)	151.7 (150.2–153.2)
	MRR		2.23 (2.13–2.33)	1.52 (1.44–1.60)	1.00	
Melanoma	ASMR	6.0 (5.5–6.5)	5.8 (5.2–6.4)	7.0 (5.8–8.3)	6.4 (5.2–7.7)	−0.6 (−1.3–0.0)
	MRR		0.89 (0.72–1.11)	1.05 (0.81–1.35)	1.00	
Prostate	ASMR	48.4 (47.0–49.7)	47.7 (45.9–49.4)	46.7 (43.2–50.2)	43.7 (40.2–47.3)	4.0 (2.1–5.7)
	MRR		1.11 (1.02–1.21)	1.06 (0.95–1.18)	1.00	
Kidney	ASMR	18.9 (18.0–19.7)	19.1 (18.0–20.2)	20.1 (17.8–22.3)	16.8 (14.7–18.9)	2.3 (1.3–3.3)
	MRR		1.15 (1.00–1.31)	1.17 (0.99–1.37)	1.00	
Bladder	ASMR	24.8 (23.8–25.8)	25.3 (24.0–26.6)	23.3 (20.8–25.7)	17.8 (15.6–20.0)	7.5 (6.6–8.4)
	MRR		1.49 (1.30–1.70)	1.34 (1.14–1.57)	1.00	
Eye, brain, central nervous system	ASMR	14.9 (14.2–15.7)	14.9 (13.9–15.9)	14.8 (13.0–16.7)	14.1 (12.3–15.9)	0.8 (0.0–1.6)
	MRR		1.06 (0.92–1.22)	1.04 (0.88–1.24)	1.00	
Non-Hodgkin Lymphoma	ASMR	14.4 (13.7–15.1)	13.9 (13.0–14.9)	15.9 (13.9–17.9)	13.2 (11.3–15.1)	0.7 (−0.2–1.7)
	MRR		1.09 (0.93–1.27)	1.23 (1.02–1.47)	1.00	
Multiple myeloma	ASMR	9.8 (9.2–10.4)	9.9 (9.1–10.7)	10.8 (9.2–12.5)	8.6 (7.1–10.1)	1.3 (0.6–2.0)
	MRR		1.12 (0.93–1.35)	1.20 (0.96–1.51)	1.00	
Leukaemia	ASMR	18.9 (18.1–19.8)	18.8 (17.7–19.9)	19.1 (16.9–21.3)	17.2 (15.0–19.4)	1.6 (0.5–2.7)
	MRR		1.14 (0.99–1.31)	1.12 (0.95–1.33)	1.00	

^a^Age-standardized mortality rate using the Belgian population at the moment of the 2001 census as reference; expressed per 100,000 men

^b^Mortality Rate Ratio

^c^Low-educated persons have completed lower secondary education or less

^d^Mid-educated persons have completed upper secondary education

^e^High-education persons have completed tertiary education

^f^Mortality rate difference

^g^The difference between the ASMR of the lowest educated and the ASMR of the highest educated

^h^95% Confidence Intervals

The majority of the cancer sites included in this study showed relative differences in mortality during the period 2001–2008. The only exceptions were cancers of the pancreas, the central nervous system, multiple myeloma, malignant melanoma and leukaemia. All *preventable* cancer sites (except for malignant melanoma) showed educational inequalities in favour of high educated men, and among these, the cancer sites amenable to *behavioural change* showed the largest relative inequalities. Mortality of cancers of the lung and head and neck showed the largest educational inequalities. For example, compared to high educated men, low educated men were about 2.2 (95% CI: 2.1–2.3) and 2.0 (95% CI: 1.7–2.2) times more likely to die from lung and head and neck cancer respectively.

### What is the recent trend in inequalities in cancer mortality in the 2000s compared to the 1990s for Belgian men?

The mortality trend favoured low educated men in most cases (Table 4), resulting in smaller absolute educational inequalities in the 2000s compared to the 1990s. For total cancer mortality, ASMRs declined by 49.4 deaths/100,000 more among the low compared to high educated men. The largest decline in educational inequalities in terms of absolute cancer mortality was observed for cancer of the lung and stomach. To the contrary, absolute mortality inequalities in cancers of the pancreas, central nervous system and colorectum increased.

**Table 4:** Trends between 1991 and 1997 and 2001–2008 in absolute and relative educational inequalities in site-specific cancer mortality among Belgian men aged 50–79 years

Cancer site	Period	AMD ^a^	PMD ^b^	PAF ^c^	RII ^d^ (95% CI) ^e^
Total cancer mortality	1991–1997	49.4	−1.43	0.28	1.88 (1.81–1.95)
	2001–2011			0.28	1.92 (1.85–1.99)
Head and neck	1991–1997	1.2	−8.67	0.32	2.35 (1.99–2.78)
	2001–2011			0.39	2.63 (2.23–3.10)
Oesophagus	1991–1997	0.3	2.38	0.14	1.34 (1.09–1.66)
	2001–2011			0.13	1.34 (1.13–1.59)
Stomach	1991–1997	13.6	23.68	0.47	3.05 (2.50–3.73)
	2001–2011			0.27	2.03 (1.66–2.47)
Colorectal	1991–1997	−1.8	−5.85	0.14	1.21 (1.08–1.36)
	2001–2011			0.20	1.24 (1.20–1.50)
Liver	1991–1997	−0.2	−0.92	−0.02	1.04 (0.82–1.30)
	2001–2011			0.03	1.06 (0.87–1.28)
Pancreas	1991–1997	−5.8	−14.68	−0.15	0.93 (0.78–1.10)
	2001–2011			−0.01	1.15 (0.99–1.34)
Lung and trachea	1991–1997	44.6	−1.70	0.47	3.18 (2.98–3.39)
	2001–2011			0.47	3.34 (3.14–3.55)
Melanoma	1991–1997	−0.4	−12.53	−0.20	0.90 (0.57–1.41)
	2001–2011			−0.07	0.79 (0.57–1.09)
Prostate	1991–1997	5.6	3.12	0.12	1.20 (1.06–1.36)
	2001–2011			0.10	1.17 (1.03–1.33)
Kidney	1991–1997	1.5	7.88	0.19	1.12 (0.89–1.40)
	2001–2011			0.11	1.16 (0.95–1.42)
Bladder	1991–1997	5.8	7.84	0.33	1.96 (1.62–2.38)
	2001–2011			0.28	1.70 (1.41–2.05)
Eye, brain, central nervous system	1991–1997	−3.2	−10.70	−0.10	0.91 (0.74–1.12)
	2001–2011			0.05	1.08 (0.87–1.34)
Non-Hodgkin Lymphoma	1991–1997	0.3	0.48	0.07	0.97 (0.77–1.23)
	2001–2011			0.08	1.02 (0.81–1.27)
Multiple myeloma	1991–1997	0.1	−0.53	0.12	1.23 (0.90–1.68)
	2001–2011			0.12	1.08 (0.83–1.42)
Leukaemia	1991–1997	−0.9	−4.69	0.03	1.10 (0.88–1.37)
	2001–2011			0.09	1.18 (0.96–1.44)

^a^Absolute Mortality Decline: Difference between low and high educated in absolute mortality decline: (ASMR_1990low_ - ASMR_2000low_) - (ASMR_1990high_ - ASMR_2000high_)

^b^Proportional Mortality Decline (in % points): Difference between low and high educated in proportional mortality decline: 100* (ASMR_1990low_ - ASMR_2000low_)/ASMR_1990low_ - 100*(ASMR_1990high_ - ASMR_2000high_)/ASMR_1990high_

^c^Population Attributable Fraction of education for mortality: (ASMR_tot_ - ASMR_high_)/ASMR_tot_

^d^Relative Index of Inequality

^e^95% Confidence Intervals

When looking at the proportional mortality decline, the picture was more diverse. Stomach cancer mortality showed the most favourable trend for the low educated men, with a stronger decrease of 23.7% points in low compared to high educated men. This favourable trend towards less inequality was also seen for mortality from cancer of the kidney, bladder, prostate and oesophagus. However, for mortality from cancers of the pancreas, the central nervous system, head and neck, colorectum and for leukaemia and malignant melanoma, educational inequalities increased. The population attributable fraction (PAF) measures the population impact of these educational inequalities. As observed in Table 4, the population impact increased for the cancer sites that showed the largest proportional mortality increase earlier. Pancreatic cancer is an interesting cancer site because the PAF varied over time from −0.15 to −0.01. This means that in the 1990s, there would have been 15% more mortality due to pancreatic cancer if everyone had the mortality rate of the high educated men, whereas in the 2000s, this was no longer the case. Moreover, the population impact of educational inequalities decreased between the 1990s and the 2000s for cancer of the stomach, kidney and bladder. For example, in the 1990s, 47% of the male stomach cancer mortality could have been avoided if the total population had the same mortality of high educated men compared to 27% in the 2000s.

All the measures above reflect trends in absolute inequalities. We also calculated the Relative Index of Inequalities (RII) for both periods to study trends in relative inequalities. For total cancer mortality as well as for the majority of cancer sites, the 95% Confidence Intervals of the RII did overlap, pointing at the absence of any significant changes over time. Stomach cancer was the only exception, showing a decreasing relative inequality with a RII that went from 3.1 in the 1990s (95% CI: 2.5–3.7) to 2.0 (95% CI: 1.7–2.5) in the 2000s (*p* = 0.005).

## Discussion

### Interpretation of the results

The results were in line with the first hypothesis, which assumed that educational inequalities would be larger and more distinct for preventable cancers. Indeed, the majority of *preventable* cancer sites showed higher mortality rates for low-educated men. In general, cancer sites amenable to *behavioural change* more often showed significant educational inequalities compared to cancer sites amenable to *medical interventions*. In Belgium, health insurance is mandatory, and covers about 99% of the total population [39]. This explains why inequalities are larger for the cancer sites related to behavioural change instead of to medical interventions.

Cancers of the lung and the head and neck showed the largest educational inequalities both in absolute and relative terms. Generally, these results are consistent with other European studies. Mackenbach et al. [16] also observed the largest relative educational inequalities in Europe in the 2000s for cancers of the head and neck, lung and oesophagus [16]. In France, the largest inequalities were observed for the same cancer sites, except for colorectal cancer characterized by modest relative inequalities [1]. Inequalities were also pronounced for cancers of the oesophagus and liver. This was in line with a study of Jemal et al. [40] covering inequalities in the 1990s and 2000s in the United States. Other European studies found similar results, with inequalities in cancers of the lung, upper aero-digestive tract, and stomach explaining a great deal of the SEP inequalities in general cancer mortality [2, 3, 41].

The second hypothesis, which assumed an increase in inequalities for preventable cancers, was not confirmed by our results. Absolute cancer mortality generally showed a trend towards less inequality. In addition, the cancers that did show an increase in mortality differences, i.e. cancer of the colorectum, pancreas, central nervous system and malignant melanoma, were only partly preventable (colorectal cancer and malignant melanoma). Stomach cancer mortality showed the largest decrease in inequality, both in relative and absolute terms. The decrease in stomach cancer mortality was larger among the low-educated groups, which points to the fact that the advancement that was made (the decline in prevalence of Helicobacter pylori infection [42] was now widespread in society [14]). Studies examining recent trends in educational inequalities for multiple cancer sites using nationwide exhaustive population data are scant to our knowledge. Nationwide studies based on sample data in France and Britain [1, 43] and on linked mortality data in Barcelona [41] also showed generally stable relative inequalities in male cancer mortality over time. However, in France, absolute inequalities declined for men [1], as observed in our study.

### Link with fundamental cause theory: Differences in resources

Educational differences in mortality reflect differences in resources [16]. Education implies knowledge resources that can be utilized to maximize health [44]. These resources include a variety of capacities such as financial means [45]; stable employment [44, 45]; health literacy [45]; being receptive to prevention messages [46]; being able to change health behaviours [46]; and making proper use of the health system [46]. Consequently, it does not come as a surprise that the cancer sites with the largest educational differences are those that are highly amenable to behavioural change (e.g. cancers of the lung or head and neck) and (to a smaller extent) cancer sites amenable to medical interventions (e.g. colorectal and prostate cancer), which is in line with the fundamental cause theory [16, 23, 24]. As educational inequalities are observed for almost all cancer sites, we can assume that there is not one single cause in terms of proximal factors that can be responsible for these inequalities [44]. Consequently, both disease risk factors and factors related to healthcare should be taken into account [44].

Low educated people are more vulnerable to unhealthy behaviours such as smoking, physical inactivity, being overweight and obese, (excessive) alcohol consumption, bad oral hygiene, risky sexual behaviour, human papillomavirus (HPV) infection and exposure to occupational agents [16, 45, 47, 48]. Interactions between these risk factors even strengthen their carcinogenic effects [41]. Low educated people are also more likely to be in bad health initially, and prevention messages about healthy habits and collective facilities (e.g. tobacco control initiatives) might have a differential impact among the social strata [44, 49]. Moreover, low educated people are more likely to have lower levels of social support and to have less control over their lives [4]. Another important risk factor for (cancer) mortality is healthcare utilization. This is especially important for cancers amenable to medical interventions, as these have a 5-year relative survival rate of more than 70% [27]. Low educated people are less likely to seek timely medical attention (causing late stage at diagnosis), and are less likely to have access to good quality healthcare [3, 16, 24, 43, 45]. Likewise, participation rates in organized screening are lower among low educated people [50]. Moreover, high educated people are more likely to be early adopters whenever new developments in disease management are made [19, 44, 50]. Taken together, low educated people might be more susceptible to new arising health threats, and hence show higher cancer incidence rates, as well as lower survival rates due to a lower ability to cope with the aggressiveness of cancer and respond to the treatment [3, 19]. Despite the almost full coverage of health insurance in Belgium, differences in health care utilization are still observed by SE group. Data of the Belgian Health Interview Survey (BHIS) prove that low educated men are more likely to smoke, to have limited physical activity, to be obese and to show excessive consumption of alcohol [51–54]. No educational gradient has been observed in the participation rate of colorectal screening [55], however, low educated Belgian men were more likely to delay medical care because of financial reasons [56].

The cancers showing (large) educational inequalities in this study are all associated with lifestyle-related factors. We will now discuss the most important ones.

Lung cancer is widely acknowledged as being caused by smoking tobacco [1–3, 5, 6, 29, 41, 50] as well as occupational exposures [3, 57, 58]. The observation that lung cancer mortality declines in all educational groups points to the fact that Belgian men went through all four phases of the smoking epidemic [20]. Head and neck cancers (oral cavity and lip, larynx and pharynx) are associated with smoking, as well as with alcohol use [1, 2, 48, 50, 59–61]. Earlier research reported that these lifestyle habits are causing 70% of head and neck cancers [48, 61], with alcohol being the most important contributor [50]. Another recently emerging risk factor for head and neck cancers (especially oropharyngeal cancer) is infection with HPV [61–64]. Yet, according to the literature, a considerable part of the burden and aetiology of cancers of the head and neck remains unexplained [48, 61]. Colorectal cancer is associated with behavioural factors such as cigarette smoking, alcohol use, physical inactivity, and excess bodyweight [41, 65, 66]. Mortality decreased in all educational groups, probably due to a healthier lifestyle [49], and to better treatment protocols [49]. Targeted screening, hereby reducing late stage at diagnosis, might also contribute to the decline [65]. Despite the overall decrease in mortality, the decline takes place at a faster pace for high educated men, resulting in increasing absolute educational inequalities. Although the BHIS did not observe educational differences in colorectal cancer screening [55], educational differences in stage at diagnosis (because of postponement of seeking medical help due to financial reasons) cannot be ignored as a possible explanation as well as differences in lifestyle factors [51–53]. Stomach cancer mortality has significantly dropped over time, yet educational differences remain. Stomach cancer is an aggressive tumour with a short survival. This suggests that the inequalities are mainly due to exposure to risk factors, rather than to differences in healthcare [41, 67]. Risk factors associated with stomach cancer are infection with Helicobacter pylori as well as smoking [41, 67]. Educational inequalities in mortality due to cancer of the bladder and oesophagus remained important. Again, smoking (for both), and alcohol use and dietary disorders (for oesophageal cancer) are likely to play a part [1, 41, 50]. Although prostate cancer mortality declined in all groups, low educated men are still worse off relative to high educated men. The most important risk factor is high age [67]. Since prostate cancer has a high survival rate, educational differences are probably related to differences in management. Advancements have been made in disease management (e.g. hormone therapy, a wider adaptation of radical prostatectomy in the elderly, prostate-specific antigen test, and radiotherapy) [67], and high educated men might be more likely to use these developments.

For the *non-preventable* cancers, inequality is rising for cancer of the pancreas and central nervous system. Although not defined as preventable, apart from old age, genetic factors and medical conditions (such as diabetes mellitus, chronic pancreatitis, or cholecystectomy), the only established risk factors are cigarette smoking (explaining only one fourth), and food consumption patterns [68, 69]. A large part of the aetiology thus remains poorly understood [69]. Pancreatic cancer is a relatively rare tumour but because of its extremely low survival rate, mortality rates are quite high [69]. Based on the assumption that innovation in the prevention and treatment of pancreatic cancer is lacking [6], we would assume the association between education and pancreatic cancer mortality not to have changed over time. However, a reversal of the association was observed, with a disproportional decrease in pancreatic cancer mortality in favour of high educated men. This trend might be related to the smoking epidemic.

### Strengths and limitations

This study evaluates (trends over time in) educational inequalities in cancer mortality. The dataset used for this study consists of a high-quality, exhaustive dataset containing all deaths during the study period. To our knowledge, such a rich source of information containing nationwide individually linked data on cancer mortality and educational attainment is unique outside the Nordic context. Moreover, through the direct individual link between census and register data, a numerator-denominator bias was eliminated. This high-quality standard of the dataset enables to give precise estimates of (trends in) the association between cancer and education at the individual level. In order to capture the full extent of inequalities and to avoid bias, both absolute and relative measures of inequality were calculated [31–34].

A limitation of this study is that the dataset only provides information on mortality, and not on cancer incidence and survival. Cancer mortality being the result of cancer incidence and cancer survival, this paper only tells part of the story [41]. Furthermore, the dataset does not contain any information on health behaviours or access to and quality of healthcare. The reasons behind the cancer mortality inequalities thus largely remain a black box (cf. infra).

SEP was operationalized using educational attainment. Related to health, education captures a person’s capacity to prevent health damage and to tackle illness through suitable care pathways [3]. Education is commonly used as an indicator for SEP and has many advantages [19]. It is available for almost everyone in the population [44–46], in contrast to job status, which is more difficult to capture for the retired and non-working population [44]. Furthermore, education is a stable indicator and has a close association with other indicators of SEP [45], such as job status and income [19]. Moreover, compared with other SEP indicators, education is less sensitive to reverse causation, as it is obtained relatively early in life [16]. A disadvantage is that it is related to both age and period [46]. Our data showed an increase in the share of the population that is highly educated. To account for the different educational distribution between both periods, RIIs were estimated [5]. However, we must bear in mind that the different educational distribution might reflect a shift in the role and significance of education which we cannot adjust for [44].

Furthermore, a transition took place in the ICD coding system between the two periods: from ICD-9 to ICD-10. This can possibly account for some of the variation in mortality rates between the periods, although its impact is probably limited since differences between the revisions are minor [2, 6, 17, 49].

## Conclusions

To sum up, educational differences in site-specific cancer mortality persist in the 2000s in Belgium, mainly for the more *preventable* cancer sites*.* This inequality is mainly due to a lower exposure to risk factors, and (to a lesser extent) a different health care utilization among higher SEP groups. According to Link and Phelan, policies should either reduce inequalities in the resources themselves or develop interventions that are more equally distributed across SEP groups [8]. In any event, reducing social inequalities should be high on the agenda of a good public health policy. Nevertheless, public health policies aiming at the general population might also entail persisting or increasing health inequalities, as lower-SEP groups might feel the public health message does not concern them [17]. Lifestyle and healthcare utilization factors need to be considered within their SEP context, both regarding disease aetiology and prevention [5, 61], whereas barriers that hamper healthy behaviours need to be removed in lower-SEP groups [5]. Future studies should preferably include larger follow-up times to monitor trends over time, because in our paper, trends in inequalities were rather stable, maybe because of the short time span used. Finally, researchers need to think about the potential role of unidentified risk factors and pathways linking SEP to cancer mortality [5, 61].

## Abbreviations

ASMR: age-standardized mortality rate
BHIS: Belgian Health Interview Survey
FCT: Fundamental Cause Theory
HPV: human papillomavirus
ICD: International Classification of Diseases and Related Health Problems
ISCED: International Standard Classification of Education
MRD: mortality rate difference
MRR: mortality rate ratio PAF population attributable fraction
RII: relative index of inequality
SE: Socioeconomic
SEP: Socioeconomic position
